# The shark flap: a modified internal mammary artery perforator flap for composite defects in head and neck reconstruction

**DOI:** 10.1080/23320885.2023.2178924

**Published:** 2023-02-15

**Authors:** Anna Scarabosio, Alessandro Tel, Filippo Contessi Negrini, Roberta Albanese, Massimo Robiony, Piercamillo Parodi

**Affiliations:** a Plastic Surgery Resident at Santa Maria della Misericordia Hospital, Udine, Italy; b Maxillo-Facial Surgery Resident at Santa Maria della Misericordia Hospital, Udine, Italy; c Plastic Surgery Consultant at Santa Maria della Misericordia Hospital, Udine, Italy

**Keywords:** iMAP flap, pedicled flap, composite flap, head and neck reconstruction, shark flap

## Abstract

This report describes a multi-vector variant of IMAP flap which allows to reconstruct composite head and neck defects. It was named the ‘shark flap’ because of its shape: a main body (the regular IMAP) and a superior ‘fin’ based on a randomic vascular pathway.

## Introduction

In the last three decades, surgical procedures for head and neck reconstruction aimed to preserve organ function and quality of life. Historically, first option is represented by local muscle flaps. Nevertheless, they do not often provide a sufficient quantity of skin islands, may result too thick for being able to achieve the physiological function of the previously debulked area and may cause substantial comorbidities for the patient [1]. To avoid these disadvantages, free flaps have been introduced. However, this kind of reconstruction requires higher technical expertise, longer operating times and increased costs. Besides, a large number of patients with head and neck cancer are not ideal candidates for free flap reconstruction because of high morbidity risk and the relevant presence of comorbidities, such as old age, frailty, malnutrition, previous radiotherapy, increased thrombotic risk [2,3].

The internal mammary artery perforator (IMAP) flap proved to be one of the most commonly used options in head and neck reconstruction for many years since it was first described by Yu in 2006 [4]. This flap consists of an adaptation of the deltopectoral flap and represents a good local option described for small-medium defects [5].

Traditional indications for the IMAP flap include reconstruction of various defects of the head, neck, and chest. Usually, the superior limit of IMAP flap is the lower third of the face[6]. This flap is based on a vessel which originates from the internal mammary artery (IMA), perforates at the inferior border of the rib cartilage and then follows a laterocaudal direction [7]. As a consequence, IMAP perforasome has a strict axial orientation and becomes suitable for multi-vector reconstruction.

According to Taylor et al. [8], vascular connections are numerous between the upper breast/epigastric internal system and the branches of the acromiothoracic axis, allowing to design a larger composite flap based on these two vascular pathways.

Applying the well-known concept of perforasome [9], the Authors searched whether an extension of the conventional skin island could be applied to the IMAP flap to solve issues related to vast defects. To support their assumptions, the Authors reported a clinical case of a patient with a large skin defect located in the cheek and right latero-cervical area which they were not able to cover rotating just a standard axial IMAP flap. A local flap from the cheek was rotated to cover the wide surgical defect and the subsequent reconstruction of this donor site and the neck was performed by applying to the standard IMAP skin design a lateral extension beyond the apparent perforasome territory, reaching the thoraco-acromial area of perfusion. This flap, whose vascular supply may be considered partially randomic but still mainly dependent from perforators of the IMAP flap, was named the shark flap because of its shape: a main body (the regular IMAP) and a superior ‘flipper’ based on a randomic vascular pathway. Potentially this variation could be applied in every area around the classic IMAP, making this a highly adaptable flap for composite and multi-vector defects. This variation is described in our clinical case and may represent a starting point for further variants.

## Case report

A 53-year-old woman presented with recurrent squamous cell carcinoma located in the cheek area, extending in depth until mandibular periosteum, through the parotid gland and masseter. She required a radical resection which included a large skin area (14 × 16 cm) of the cheek, a total parotidectomy and the resection of the zygomatic arch and the coronoid process, down to the level of the temporomandibular joint (TMJ) capsule. Fronto-temporal and buccal branch of the right VII cranial nerve were sacrificed to ensure safe margins. A prophylactic supraomohyoid neck dissection was performed during the same surgery.

Reconstruction first involved a joint capsuloplasty and a deep temporal fascia rotation flap for TMJ coverage . The cranial skin defect was covered by rotating a flap from the bottom part of the cheek. After rotating the flap, the donor site defect was contiguous with the neck incisions for the dissection.

**Figure 1.**
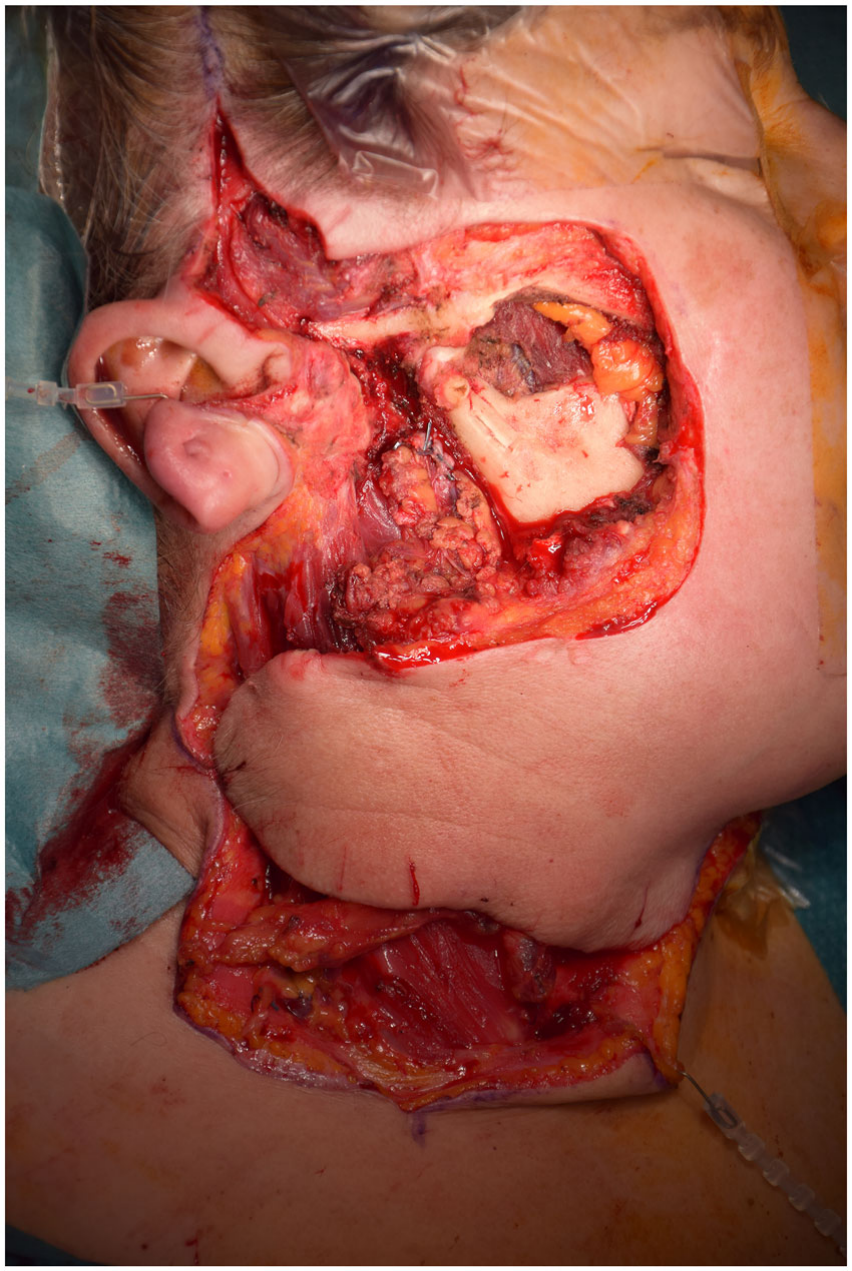
Radical resection residual defect and cheek flap which is rotating to restore the cheek area.

Restoring the skin surface represented a great challenge in this case because of the extent of the residual wound and the relative stiffness of the surrounding tissues (Figure 1).

In the Author’s opinion the most suitable solution appeared to be an IMAP flap. Traditionally it is a fasciocutaneous axial flap based on the internal mammary artery perforators which originate at first to fifth intercostal spaces. Preoperatively, perforator vessels were studied using Color-Doppler Ultrasound (CDUS) selecting the perforator with the largest caliber [7]. Its use allowed to design the flap on the pre-marked dominant perforator which resulted to be the one at the second intercostal space. Differently from the standard so-called ‘second IMAP’, the shark flap was strictly designed on the main perforator course thank to the use of CDUS. The skin paddle surface resulted of 176 cm^2^, which is about 25 cm^2^ larger than average second IMAP perfusion area [7,10].

If the conventional IMAP flap had been performed the entire defect would have not been covered, leaving the medial neck area unrestored. To solve this issue, the Authors decided to incorporate within the IMAP a narrower flap with a triangular shape and a random-based vascularization pattern with the possibility to be transposed anteriorly to fill the residual wound between the inferior margin of the rotated cheek flap and the superior margin of the neck skin.

A customized intraoperative skin paddle was drawn, and dissection was carried out in a lateral-to-medial fashion maintaining a supra-fascial plane. During dissection, great care was taken to stay at 2 cm from the lateral border of the sternum performing a gentle microvascular identification of vascular pedicle to avoid its injury [4,7,11]. The perforator was not directly visualized; an adipose tissue cuff was preserved around it to reduce pedicle kinking and twisting risk.

**Figure 2.**
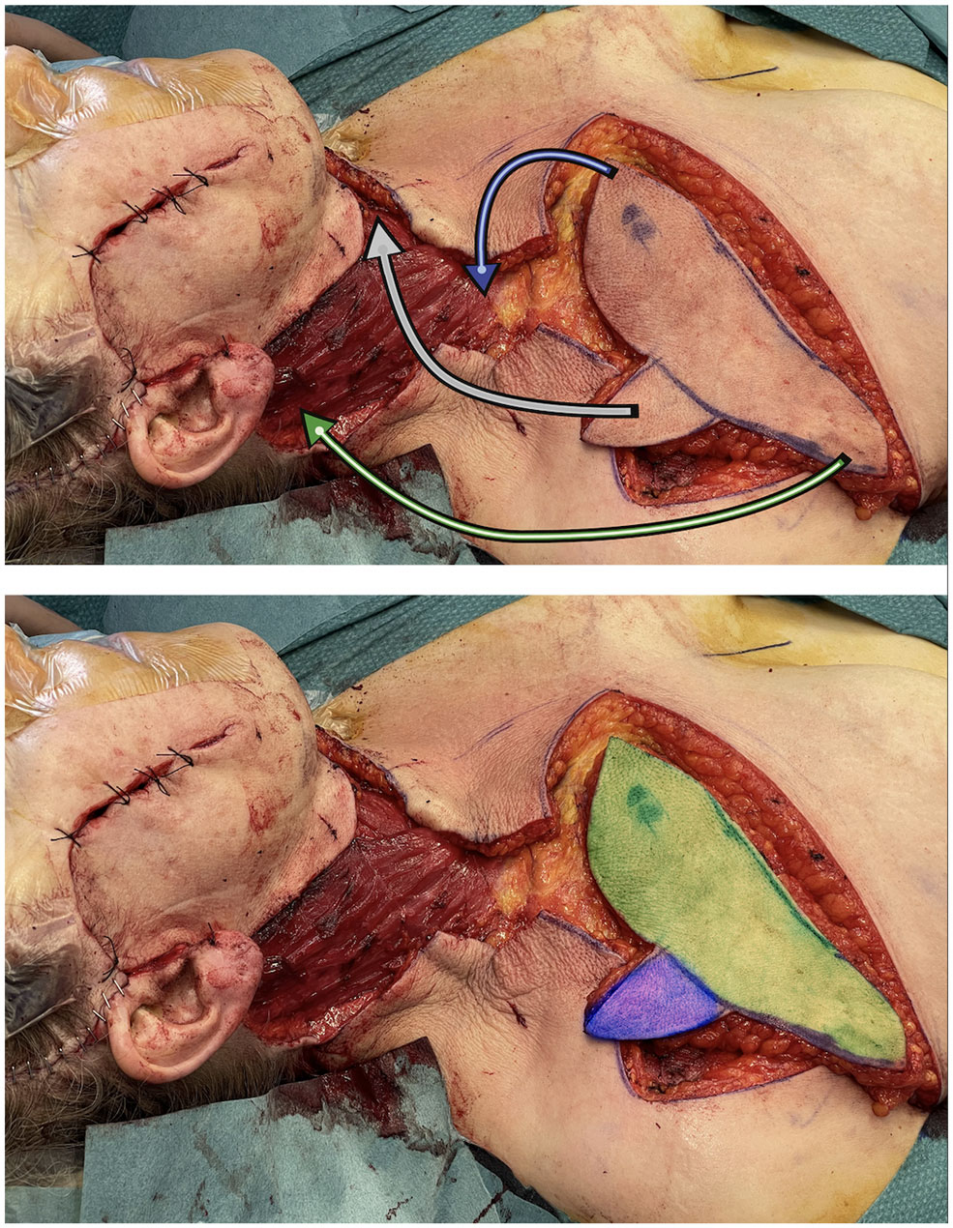
In the first picture the shark flap was harvested and just before insetting phase. Arrows are showing the trasposition movements from donor to recipient site. In the second one the two main vascular area were highlighted – green: standard IMAP flap; blu: random area base on connections between TAP and IMAP.

**Figure 3.**
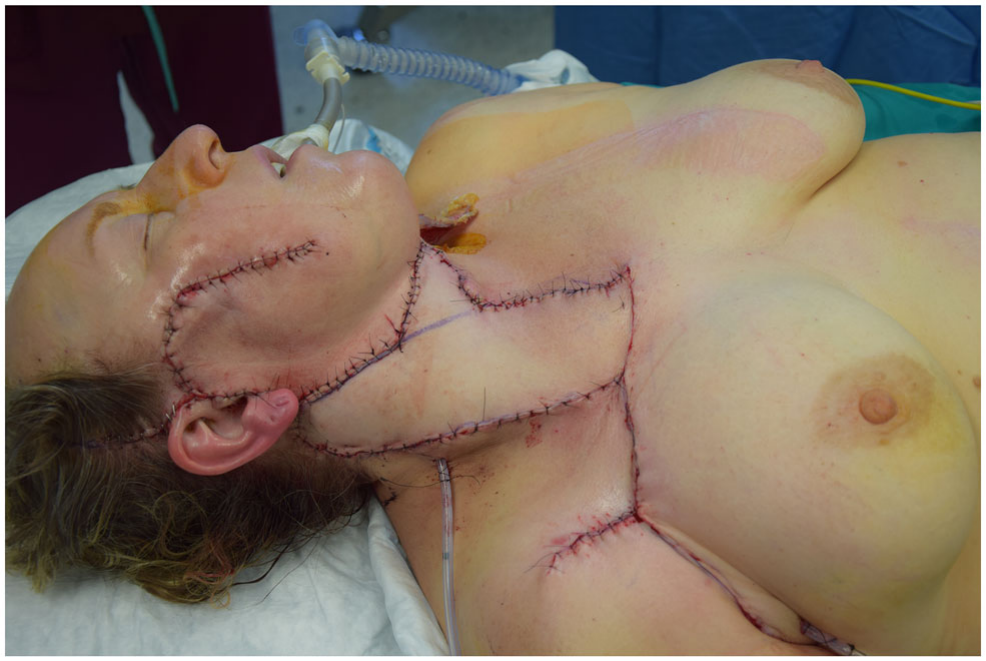
Immediate post-operative.

At the end of dissection time no venous congestion of the flap occurred, allowing to rotate it of 90 degrees to restore entirely the skin surface of the neck [12]. Therefore it may be fully considered a propeller flap. The donor area of the chest was sutured directly. The ‘fin’ of the shark flap was rotated clockwise in the safest direction and sutured to wound margins to close the defect (Figures 2 and 3). The flap was then accurately sutured following the natural linings of the patient, in particular the ‘fin’ was place right underneath the chin perpendicularly to the major axis of the neck. This placement guaranteed a tension-free closure. Post-operatively neck mobility was fully limited and a light hyperextension was maintained for almost a week, then gradually restored. In two weeks time the patient had no mobility restrictions.

**Figure 4.**
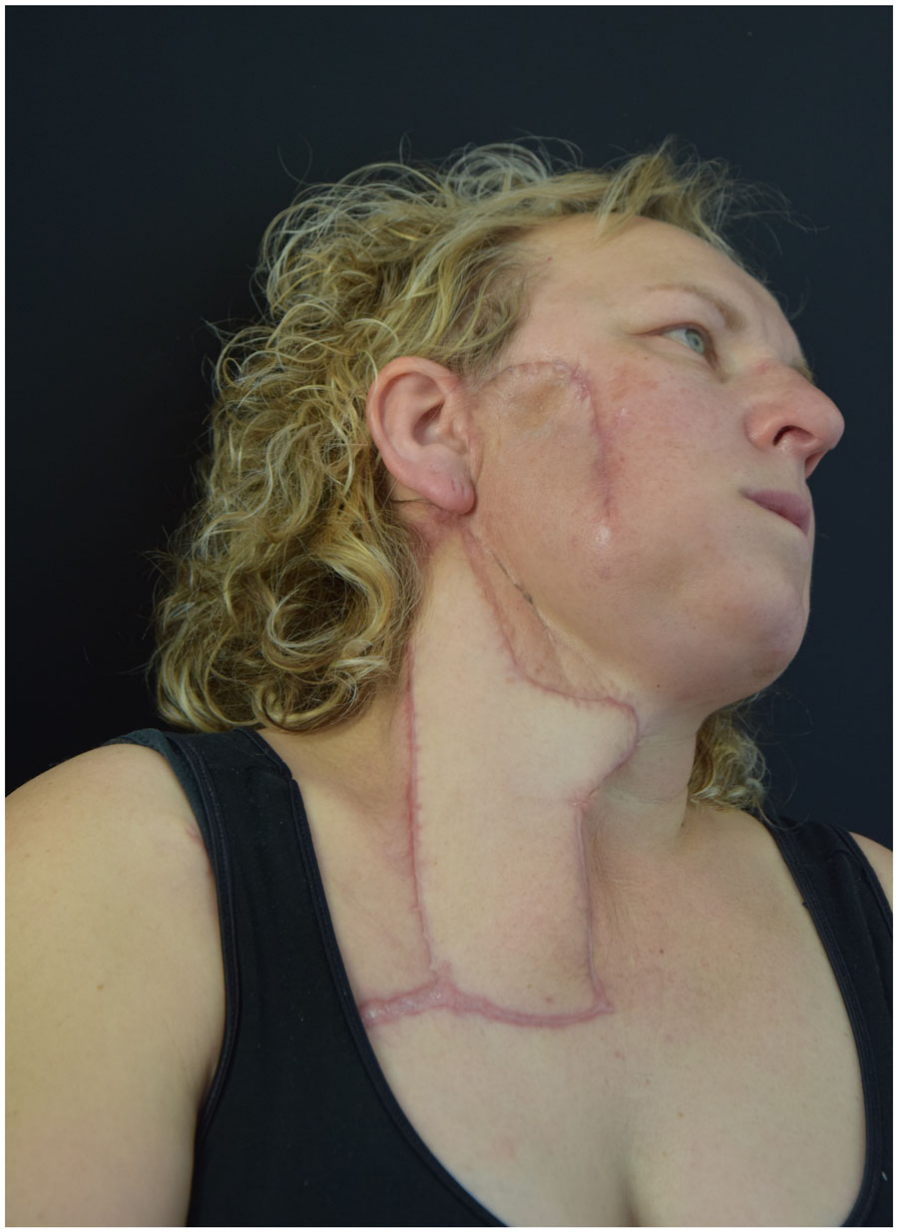
4-month follow-up - no local complications and acceptable aesthetic result.

At 4-month postoperatively the patient underwent a follow-up MR without any radiological sign of persistence of disease (Figure 4). At 6-month follow-up there was no evidence of complications, wound dehiscence or pathological scarring. There were no limitations in neck mobility. There was no clinical sign of disease relapse.

## Discussion

The presented case sheds light on relevant surgical aspects which are only marginally present in current literature. In this case we are able to find two interesting topics: the combination of a randomic area to axial IMAP flap and the simultaneous association with a local cheek flap. For head and neck reconstruction, the use of local flaps can avoid significant morbidity related to the harvesting site and decrease intraoperative complications related to the prolonged surgical time required for a microsurgical intervention, as well as the risk of failure. Among local flaps, the IMAP was proven to be a reliable, thin and pliable flap with a suitable arc of rotation and perfect color match for head and neck [13].

Moreover, the IMAP flap leads to an acceptable aesthetic result and low donor site morbidity. Its constant and reliable vascularization allows to design small to medium sized flaps; on the other hand, its arrangement as an axial flap limits the design especially in its transverse diameter. Furthermore, it may require costal cartilage resection when pedicle is short; it can affect cosmetic outcome in women as retracting scars can lead to breast asymmetry and nipple distortion; distal flap necrosis can occur if the skin paddle is extended to the deltoid region; and hair growth in hirsute skin in men [14]. To date there are no specific studies on its angiosome and the possibilities of harvesting the flap beyond the anterior axillary line is considered risky for its survival. Nevertheless the author’s variant of the IMAP is based on the vascular study of Taylor et al. in which an dense vascular network is demonstrated between the upper breast/epigastric internal system and the branches of the acromiothoracic axis [8]. This has also been supported by Saint-Cyr et al. in 2008 where the second IMAP clearly vascularized a superolateral area usually supplied by the acromiothoracic vessels [14]. Known connections between high-flow vascular systems could increase the possibility of extending the ‘classic IMAP angiosome’. In particular, if the anterior axillary pillar represents the lateral limit [7], beyond which the risk of necrosis is high; little is known of the extension in its transverse diameter. Favorable flap design such as bilobed flap or trilobed flap could somehow facilitate the coverage of composite defects.

These variants could extend IMAP indications, including larger and composite defects. Moreover, clockwise rotation it as a propeller flap allows to consider it for more distant defects.The Indocyanine Green (ICG) angiography at the harvesting time would add more information about the complete angiosome.

The use of local flaps, mobilized through long-pedicled flaps, provides several advantages, including the aforementioned reduction in morbidity, a close-looking texture pattern and a faster harvesting phase. Looking at this IMAP variant on the aesthetic point of view there can be found two further advantages. Even if it necessarily leaves more scars, in this specific clinical case the Authors were able to perform a direct closure of the donor site, without using a skin graft. Moreover, the second lobe scar of the shark flap stayed in the anterior axillary pillar, being easily hidden by the axillary fold.

This preliminary report presents new strategies to the reconstruction of complex head and neck defects which can be treated by using composite design flaps. The chance to be able to customize a secondary, random-based area, perpendicularly oriented to the main vascular axis of the IMAP, may be useful particularly in head and neck because of its spatial complexity. The Authors suggest that this type of design can be considered reliable, reproducible and scalable for many types of reconstruction of the head and neck region. In addition, the supplementary, random-based, skin paddle attached to the IMAP flap extends reconstructive possibilities for this technique even for defects of larger size and located more medially. However, a more specific vascular study of the anatomic angiosome performed on higher numbers will be necessary to support this idea and define the underlying anatomical principles. In addition, it has surely to be mentioned that this was not the only viable strategy to cover this defect. Other options may be: first the submental flap which would enable the reconstruction of the initial defect avoiding the utilization of a second flap; second, the supraclavicular flap and, finally, the anterior supraclavicular artery perforator (a-SAP) flap based on the anterior supraclavicular vessels.

## Conclusion

Nowadays thanks to perforasome concept a reconstructive surgeon is able to combine pedicled, random and free flaps to obtain the most appropriate reconstruction in terms of skin color, flap thickness and function. This case report is an example of how versatile can be this combination of different techniques. A greater number of cases and necessary vascular studies are required to build higher evidence on this interesting topic.
